# Searching for Genomic Region of High-Fat Diet-Induced Type 2 Diabetes in Mouse Chromosome 2 by Analysis of Congenic Strains

**DOI:** 10.1371/journal.pone.0096271

**Published:** 2014-05-01

**Authors:** Misato Kobayashi, Tamio Ohno, Kunio Ihara, Atsushi Murai, Mayumi Kumazawa, Hiromi Hoshino, Koichiro Iwanaga, Hiroshi Iwai, Yoshiki Hamana, Mikako Ito, Kinji Ohno, Fumihiko Horio

**Affiliations:** 1 Department of Applied Molecular Biosciences, Graduate School of Bioagricultural Sciences, Nagoya University, Nagoya, Aichi, Japan; 2 Division of Experimental Animals, Center for Promotion of Medical Research and Education, Graduate School of Medicine, Nagoya University, Nagoya, Aichi, Japan; 3 Center for Gene Research, Nagoya University, Nagoya, Aichi, Japan; 4 Division of Neurogenetics, Center for Neurological Diseases and Cancer, Nagoya University Graduate School of Medicine, Nagoya, Aichi, Japan; Monash University, Australia

## Abstract

SMXA-5 mice are a high-fat diet-induced type 2 diabetes animal model established from non-diabetic SM/J and A/J mice. By using F2 intercross mice between SMXA-5 and SM/J mice under feeding with a high-fat diet, we previously mapped a major diabetogenic QTL (*T2dm2sa*) on chromosome 2. We then produced the congenic strain (SM.A-*T2dm2sa* (R0), 20.8–163.0 Mb) and demonstrated that the A/J allele of *T2dm2sa* impaired glucose tolerance and increased body weight and body mass index in the congenic strain compared to SM/J mice. We also showed that the combination of *T2dm2sa* and other diabetogenic loci was needed to develop the high-fat diet-induced type 2 diabetes. In this study, to narrow the potential genomic region containing the gene(s) responsible for *T2dm2sa*, we constructed R1 and R2 congenic strains. Both R1 (69.6–163.0 Mb) and R2 (20.8–128.2 Mb) congenic mice exhibited increases in body weight and abdominal fat weight and impaired glucose tolerance compared to SM/J mice. The R1 and R2 congenic analyses strongly suggested that the responsible genes existed in the overlapping genomic interval (69.6–128.2 Mb) between R1 and R2. In addition, studies using the newly established R1A congenic strain showed that the narrowed genomic region (69.6–75.4 Mb) affected not only obesity but also glucose tolerance. To search for candidate genes within the R1A genomic region, we performed exome sequencing analysis between SM/J and A/J mice and extracted 4 genes (*Itga6, Zak*, *Gpr155,* and *Mtx2*) with non-synonymous coding SNPs. These four genes might be candidate genes for type 2 diabetes caused by gene-gene interactions. This study indicated that one of the genes responsible for high-fat diet-induced diabetes exists in the 5.8 Mb genomic interval on mouse chromosome 2.

## Introduction

The number of people with type 2 diabetes is rising worldwide and reached more than 371 million in 2012 [1]. Changes in lifestyle, including dietary changes and reduced physical activity, are associated with the increase in the number of diabetic subjects. The development of type 2 diabetes is caused by an interaction between genetic and environmental factors. To enhance prevention/intervention in type 2 diabetes, our group has focused on the interaction between dietary (environmental) factors and genetic factors. Since genetic and dietary factors can be strictly controlled when inbred animal models are used, we aim to isolate diabetogenic genes from such models feeding a high-fat diet.

**Figure 1:**
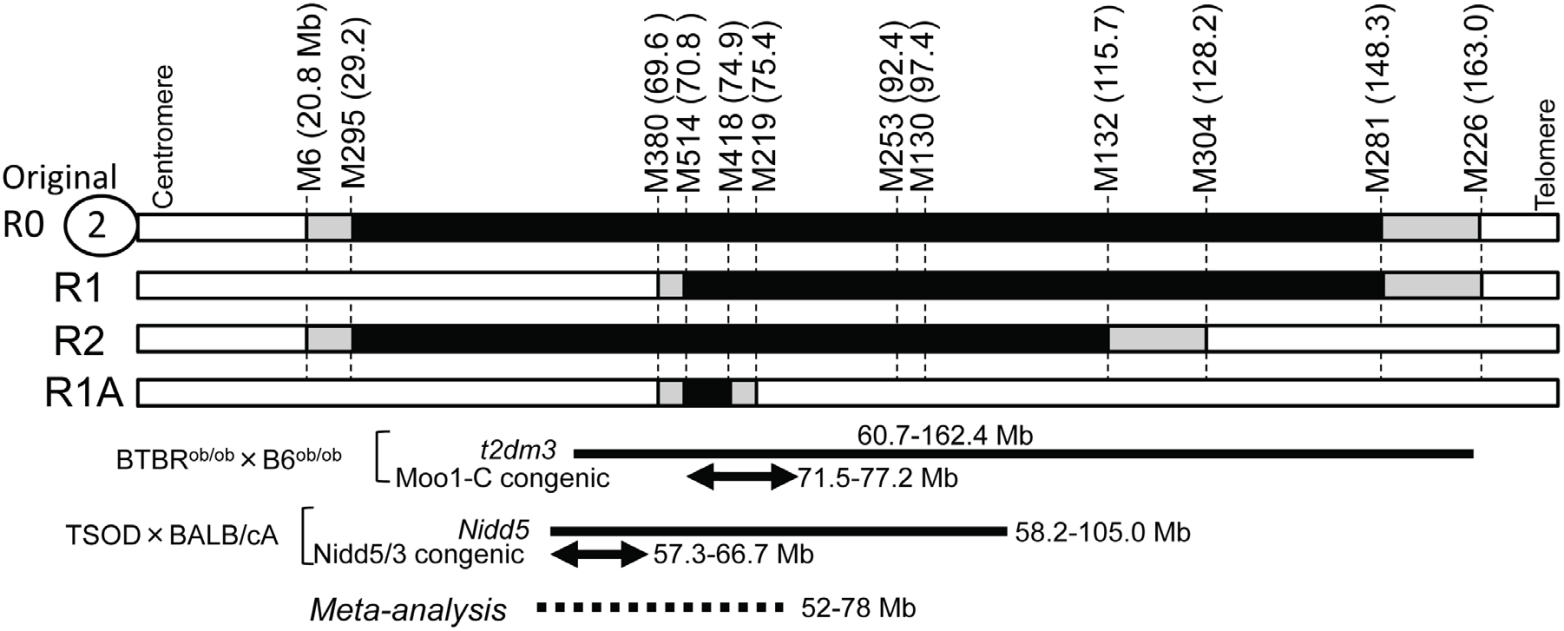
The genomic construct of congenic lines used to narrow *T2dm2sa* QTL location. The genome of each line has an SM/J background (white boxes) and was replaced by donor A/J genomic intervals (black boxes). Gray boxes show that it is not clear whether the genomic intervals were derived from A/J or from SM/J mice. Two bold solid lines indicate the genomic intervals of QTLs (*t2dm3* and *Nidd5*) for type 2 diabetes in BTBR mice [6], [7] and TOSD mice [9], respectively. Two arrows indicate the genomic regions affecting the diabetic phenotypes, which were confirmed by using Moo-C [7], [8] or Nidd5/3 [10] congenic strains. The dotted line indicates the genomic interval detected by meta-analysis for the diabetes-related QTLs of rodents [12].

The mouse SMXA-5 strain is an model for high-fat diet-induced type 2 diabetes; SMXA-5 mice develop mild obesity, impaired glucose tolerance, insulin resistance, and fatty liver [2]. The SMXA-5 strain is one of the 26 SMXA recombinant inbred strains that have been established from non-diabetic parental strains SM/J and A/J [3]. These facts show that the combination of diabetogenic genes in the non-diabetic SM/J and A/J genomes induces the diabetic phenotype in SMXA-5 mice. We previously mapped a major diabetic QTL:*T2dm2sa*, whose affective allele was A/J, on mouse chromosome 2 by using an F2 intercross between SM/J and SMXA-5 mice under feeding with a high-fat diet [4]. To verify the function of the responsible locus (*T2dm2sa*) mapped, we chose congenic mapping as a subsequent strategy. We previously constructed the original congenic strain (SM.A-*T2dm2sa,* R0), which carries an A/J-derived genomic interval (142.2 Mb) on the SM/J background (Figure 1) [4]. Phenotypic analysis of this congenic strain demonstrated that responsible gene(s) for impaired glucose tolerance and obesity exist in the genomic interval (142.2 Mb) between *D2Mit6*-*D2Mit226*. Moreover, it appeared that a combination of *T2dm2sa* and other diabetogenic loci was required for the development of high-fat diet-induced type 2 diabetes in SM.A-*T2dm2sa* mice [4]. Several hundred genes exist within this 142.2 Mb region on chromosome 2, and thus we can hardly extract candidate genes for *T2dm2sa*.

In this study, to narrow the genomic interval in which diabetogenic gene(s) exists, we newly developed three congenic strains. As a result, we succeeded in narrowing the genomic interval in which one of diabetogenic genes exists, and identified 4 genes possessing a non-synonymous SNP between SM/J and A/J mice as candidate genes within the narrowed genomic interval (5.8 Mb) by exome sequencing.

## Materials and Methods

### Animals

The diabetogenic locus (*T2dm2sa*), the development of the SM.A-*T2dm2sa* congenic mouse strain, and the donor A/J genomic interval (*D2Mit295*-*D2Mit281*) in the recipient SM/J background were previously described [4]. The R1 and R2 congenic strains were produced from original congenic SM.A-*T2dm2sa* (R0) mice (Figure 1). The R1A congenic strain was produced from R1 congenic mice. Briefly, male R0 or R1 congenic were mated to female SM/J mice to produce N1 mice (heterozygous R0 or R1 mice), and then the N1 mice were backcrossed to SM/J mice to obtain mice carrying new genomic intervals. In this study, only male mice were used for the phenotypic analyses. Mice were maintained in a temperature-controlled room (23±2°C) and 55±5% humidity with a 12-h light/dark cycle and ad libitum access to food and water under conventional conditions. Until 6 weeks of age, all mice were fed a standard laboratory chow (Labo MR Breeder, Nihon NOSAN, Japan). Animal care and all experimental procedures were approved by the Animal Experiment Committee, Graduate School of Bioagricultural Sciences, Nagoya University (approval nos. 2007050903, 2009031101, 2013021801), and were conducted according to the Regulations on Animal Experiments of Nagoya University.

### Experimental Schedule and Diet Composition

In Experiment 1 (Exp.1) using SM/J, mice of the original congenic R0, R1 and R2 strains were kept at four animals per cage and fed a powdered high-fat diet from 6 to 17 weeks of age. The powdered high-fat diet composition (weight %) was as follows: casein, 20.9; carbohydrate (corn starch: sucrose, 1∶1), 36.9; AIN93MX mineral mixture, 3.5; AIN93VX vitamin mixture, 1.0; choline chloride, 0.2; corn oil, 3.5; lard, 30.0; cellulose (AVICEL type FD-101; Asahi Chemical Industry, Osaka, Japan), 4.0. The content of fat in this high-fat diet was 33.5% (weight %). When R1A congenic mice were kept at four animals per cage and fed a powdered high-fat diet, they could not adapt to these conditions, and fought and injured each other. Therefore, in Experiment 2 (Exp. 2) using SM/J, R0 original congenic, and R1A congenic strains, mice were kept at one animal per cage and fed high-fat pellets of approximately the same composition as in Exp. 1 from 6 to 17 weeks of age. The high-fat pellet composition (weight %) was as follows: casein, 20.9; carbohydrate (corn starch: sucrose: maltodextrin 10, 94∶100∶175), 36.9; mineral mixture, 3.5; vitamin mixture, 1.0; choline bitartrate, 0.2; corn oil, 3.5; lard, 30.0; cellulose, 4.0. After 11 weeks on the high-fat diet, the mice were killed by decapitation, and the serum, liver, and fat pads were collected at 10∶00–12∶00 hours after 1-hour diet deprivation.

### Intraperitoneal Glucose Tolerance Test (IPGTT) and Body Mass Index (BMI)

After 10 weeks on the high-fat diet, IPGTT and the measurement of BMI were performed as in our previous report [4]. After 14 h of fasting (from 19∶00 to 09∶00 h), blood samples were collected from the tail vein (fasting blood glucose sample). Then, a 20% glucose solution was injected intraperitoneally (2 g glucose/kg body weight). Blood samples were collected 30, 60 and 120 min after the injection. The blood glucose concentration was measured by a glucose oxidase method (Glucose-B test Kit; WAKO, Tokyo, Japan). The AUC (area under the curve) was calculated according to the trapezoid rule from the glucose measurements at fasting, 30, 60 and 120 min (mg/dLmin). BMI was calculated as body weight (g) divided by the square of the anal-nasal length (cm).

### Serum Insulin and Lipids

Serum insulin, triglyceride, and cholesterol concentrations were measured at the end of each experiment (after feeding the high-fat diet for 11 weeks) by using a Mouse Insulin ELISA kit (Morinaga Institute of Biological Sciences Inc., Japan), Triglyceride-E kit (WAKO Pure Chemical Industries, Japan), and Cholesterol-E kit (WAKO Pure Chemical Industries), respectively.

### Exome Capture Sequencing Analysis

Genomic DNA was isolated from the tail using a DNeasy Blood and Tissue kit (Qiagen). Exome capture, the enrichment of exonic regions of genomic DNA, of SM/J and A/J were performed using a SureSelectXT Mouse All Exon kit (for AB SOLiD; Agilent Technologies) covering 49.6 MB (1.82%) of the mouse genome. We sequenced 50 base pairs of each tag in a single direction using a quarter of a cell of the SOLiD 4 system (Life Technologies) for each sample. Single nucleotide variants (SNVs) and indels (small insertions or deletions) were called by Avadis NGS with default parameters and detected 264,617 (SM/J) and 200,130 (A/J) SNVs/indels. SNVs and indels were compared to dbSNP Build 132. There were 623 exons in 63 genes between *D2Mit380* at chr2: 69617675 (mm10) and *D2Mit219* at chr2: 75416850 (mm10). The mean coverages of these exons were 32.9 for A/J strain and 24.9 for SM/J strain. The median coverages were 26 for A/J strain and 20 for SM/J strain. Nucleotide to nucleotide coverage counts revealed that 3.1% for A/J strain and 3.8% for SM/J strain were not covered by any reads. Exome data were deposited in DDBJ Sequence Read Archive (Accession No. DRA002145).

### Statistical Analysis

One-way ANOVA and subsequent Tukey-Kramer’s multiple comparison tests were used to compare the means among strains. Differences with *P*<0.05 were regarded as significant. General statistical analyses were also performed using StatView version 5.0 software (SAS Institute, Cary, NC).

## Results

### Phenotypic Analysis of R1 and R2 Congenic Mice (Exp. 1)

**Figure 2:**
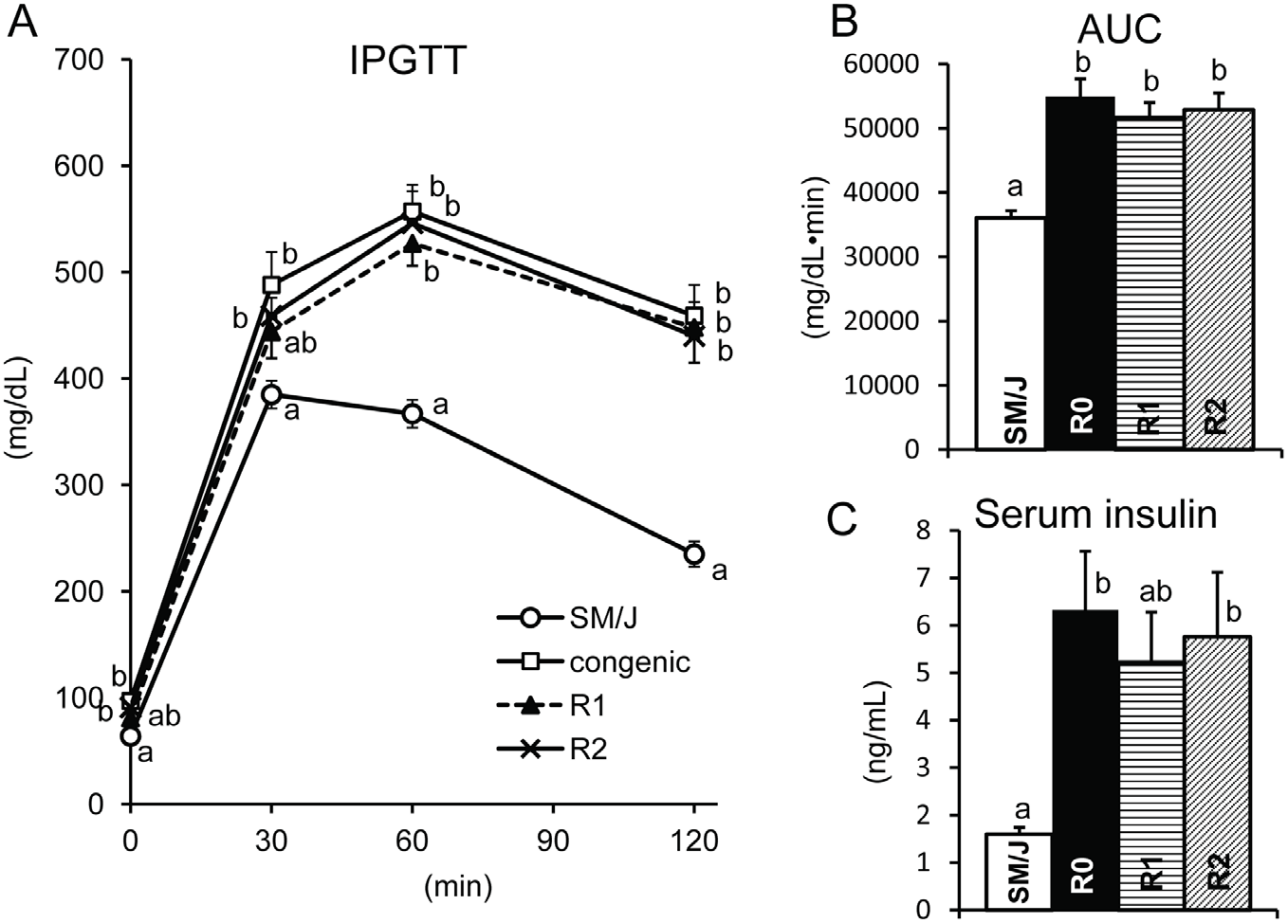
Diabetes-related traits in SM/J, original congenic R0, and congenic (R1, and R2) strains. A. The blood glucose concentrations and B. AUC during the glucose tolerance test, and C. serum insulin concentrations. ^abc,^Means not sharing a common superscript letter were significantly different among SM/J, original congenic (R0), R1, and R2 congenic strains by Tukey-Kramer test.

To explore the genomic region containing responsible gene(s) for *T2dm2sa*, we compared phenotypes of R1 and R2 congenic mice with those of SM/J and R0 congenic mice fed a high-fat diet (Table 1 and Figure 2). The body weight and BMI in R1 and R2 mice were higher than those in SM/J, a background strain (Table 1). The white adipose tissue weights (epididymal fat, mesenteric and retroperitoneal fat) were higher in R1 and R2 mice than in SM/J mice. These traits of R1 and R2 mice were similar to those of R0 mice. In the glucose tolerance test, R0, R1, and R2 mice showed clearly impaired glucose tolerance (Figure 2A). The AUC during IPGTT, a comprehensive parameter measuring the change of blood glucose concentrations, was significantly higher in R0, R1, and R2 mice than in SM/J mice (Figure 2B). In addition, the serum insulin concentration was higher in R0 (6.32±1.24 ng/ml) and R2 mice (5.75±1.36 ng/ml) than SM/J mice (1.60±0.16 ng/ml) (Figure 2C). In R1 mice, the concentration (5.22±1.06 ng/ml) tended to be higher than that in SM/J mice. The hyperinsulinemia in the R0, R1, and R2 congenic mice implies that those congenic mice have greater insulin resistance compared to SM/J mice. These results indicated that each interval of R1 and R2 contained responsible gene(s) for obesity and impaired glucose tolerance. Moreover, it is strongly suggested that responsible gene(s) exists in the genomic region overlapped (*D2Mit380*-*D2Mit304,* 58.7 Mb interval) between R1 and R2 congenic mice.

**Table 1:** Body weight, body mass index, and tissue weights in SM/J, R0, R1, and R2 mice fed a HF diet for 11 weeks.

	SM/J (n = 21)	R0 (n = 13)	R1 (n = 10)	R2 (n = 16)
Body weight (g)	24.0±0.5^a^	29.8±0.6^b^	29.8±0.7^b^	29.4±0.6^b^
Body mass index (g/cm^2^)	0.267±0.003^a^	0.313±0.005^b^	0.312±0.006^b^	0.305±0.006^b^
Non-fasting blood glucose (mg/dL)	159±4^a^	193±6^b^	182±8^ab^	179±11^ab^
Tissue weight (g/100 g body weight)				
Subcutaneous fat	2.78±0.13^a^	4.12±0.22^b^	3.58±0.22^ab^	3.58±0.18^b^
Epididymal fat	4.02±0.11^a^	5.38±0.21^b^	5.03±0.18^b^	5.24±0.19^b^
Mesenteric and Retroperitoneal fat	2.04±0.06^a^	2.58±0.08^b^	2.81±0.12^b^	2.55±0.07^b^
Liver	4.01±0.08	4.13±0.13	4.17±0.13	4.13±0.10

ab Means not sharing a common lowercase letter are significantly different by Tukey-Kramer’s test (*P*<0.05).

### Phenotypic Analysis of R1A Congenic Mice (Exp. 2)

**Figure 3:**
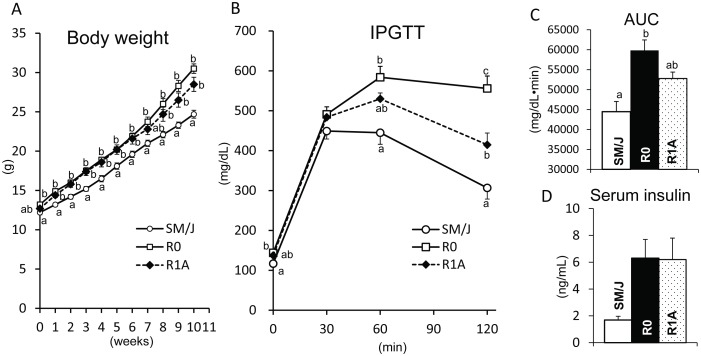
Diabetes-related traits in SM/J, original congenic R0, and congenic R1A strains. A. The body weight, B. blood glucose concentrations and C. AUC during the glucose tolerance test, and serum insulin concentrations under a high-fat diet feeding.^ abc,^Means not sharing a common superscript letter were significantly different among SM/J, R0, and R1A congenic strains by Tukey-Kramer test.

To narrow the genomic region in which diabetogenic gene(s) exists, we constructed R1A congenic mice (Figure 1) derived from the R1 congenic strain, and compared the diabetic phenotypes among SM/J, R0, and R1A (Table 2, Figure 3). The initial body weight did not differ between SM/J and R1A mice. However, after 1 week of feeding with a high-fat diet, the body weight in R0 or R1A mice was significantly higher than that in SM/J mice (Figure 3A). After feeding with a high-fat diet for 11 weeks, the BMI in R0 or R1A mice was significantly higher than that in SM/J, because the white fat pad weights of R0 or R1A mice were higher compared to SM/J (Table 2). The growth curve and obesity of R1A mice were comparative to those of R0 congenic mice. The food intakes at 3, 7, and 11 weeks of the experiment were not different between SM/J and R1A mice. In IPGTT, R0 mice showed remarkably impaired glucose tolerance compared with SM/J mice (Figure 3B and 3C). R1A mice also showed significantly higher blood glucose concentration at 120 min during IPGTT than that in SM/J mice (Figure 3B). However, the impaired glucose tolerance of R1A was milder compared to R0 mice (Figure 3B and 3C). Both R0 and R1A mice showed equivalent hyperinsulinemia (Figure 3D). Serum TG and TC concentrations in R1A mice were not different from the respective values of SM/J mice (Table 2). These results show that at least one of responsible genes for diabetes and obesity exists in a 5.8 Mb genomic interval (*D2Mit380*-*D2Mit219*, 69.6–75.4 Mb) of R1A mice.

**Table 2:** Body mass index, food intake, serum lipids, and tissue weights in SM/J, R0, and R1A congenic mice fed a HF diet for 11 weeks.

	SM/J (n = 12)	R0 (n = 10)	R1A (n = 12)
Body mass index (g/cm^2^)	0.255±0.004^a^	0.301±0.004^b^	0.284±0.008^b^
Food intake (g/day) at 3 wks	2.43±0.11^a^	3.05±0.07^b^	2.85±0.21^b^
Food intake (g/day) at 7 wks	2.87±0.20	3.58±0.06	3.19±0.19
Food intake (g/day) at 11 wks	2.89±0.17	3.53±0.12	3.28±0.25
Non-fasting blood glucose (mg/dL)	171±4^a^	182±4^ab^	184±8^b^
Serum TG concentration (mg/dL)	203±9	254±25	245±12
Serum TC concentration (mg/dL)	187±3	177±13	169±10
Tissue weight (g/100 g body weight)			
Subcutaneous fat	2.43±0.22^a^	3.61±0.14^b^	2.98±0.18^b^
Epididymal fat	3.29±0.27^a^	5.21±0.22^b^	4.57±0.22^b^
Mesenteric and Retroperitoneal fat	2.00±0.16^a^	2.80±0.14^b^	2.38±0.12^b^
Liver	4.33±0.10	4.53±0.13	4.49±0.11

TG, triglyceride; TC, total cholesterol.

ab Means not sharing a common lowercase letter are significantly different by Tukey-Kramer’s test (*P*<0.05).

### Candidate Genes with Non-synonymous Coding SNPs Determined by Exome Sequencing

In Exp. 2, we narrowed the genomic region containing one or more of responsible genes to 5.8 Mb, in which 63 genes exist. We previously performed DNA microarray analyses in liver, skeletal muscle, and epididymal fat of SM/J and R0 congenic (data not shown). In R1A region between 69.6 Mb and 75.4 Mb on chromosome 2, there was no gene whose expression level was different (fold change >2.0 or <0.5) between SM/J and R0 mice. We could not extract candidate genes which affect “quantity” of gene expression. Therefore, in order to extract potent candidate genes which affect “quality” of protein, we next performed exome sequencing of 63 genes with a next-generation sequencer (Table S1 and S2). As a result, we identified the genes containing protein-altering variants (non-synonymous single base substitutions) between SM/J and A/J mice within R1A region (69.6–75.4 Mb) (Table 3). By exome sequencing, there were six non-synonymous SNPs with missense mutations, but no SNPs with nonsense or frameshift mutations. Subsequently, we confirmed the four non-synonymous coding SNPs by the Sanger method with a capillary sequencer (Table 3). We identified non-synonymous SNPs between SM/J and A/J in *Itga6* (integrin alpha 6), *Zak* (leucine-zipper and a sterile-alpha motif kinase), *Gpr155* (G protein-coupled receptor 155), and *Mtx2* (metaxin 2). The SNPs of *Itga6* (c.3123C>G), Zak (c.1806A>G), *Gpr155*(c.973A>G), and *Mtx2*(c.961T>C) lead to amino acid substitutions of Leu975Val, Asn518Ser, Ile188Val and Trp257Arg, respectively. We also checked the genotypes for A/J and SM/J strains between *D2Mit380*-*D2Mit219* (69.6 Mb-75.4 Mb) from Imputed Mouse SNP Resource in the Center for Genome Dynamics and validated the non-synonymous SNP at 71684254 position in *Itga6* gene, but not non-synonymous SNPs in Zak, Gpr155, Mtx2 genes (http://csbio.unc.edu/imputation/)[5].

**Table 3:** The genes identified non-synonymous mutations in the R1A region (Chr. 2: 69.6–75.4 Mb) by exome sequencing.

Location (bp)	Gene symbol	Nucleotide position	Exon	A/J	SM/J	AA^a^ position	A/J-AA	SM/J-AA	db SNP	Residue among mammals^b^
71684254	*Itga6*	c.3123	16	GTG	CTG	975	Val	Leu	rs13464795	Mu:L, Rat:V, Ho:M, Bos:V
72276260	*Zak*	c.1806	19	AAT	AGT	518	Asn	Ser	rs27970273	Mu:N, Rat:S, Ho:S
73211964	*Gpr155*	c.973	4	ATC	GTC	188	Ile	Val	rs224181081	Mu:I, Rat:I, Ho:I, Bos:I
74714508	*Mtx2*	c.961	10	TGG	CGG	257	Trp	Arg	rs27967689	Mu:W, Rat:R, Ho:R, Bos:-

a AA means amino acid.

b Mu (*Mus musculus* reference residue in C57BL/6), Rat (*Rattus norvegicus*), Ho (*Homo sapiens*), Bos (*Bos taurus*).

## Discussion

Previously, by using an original congenic strain (SM.A-*T2dm2sa*, R0), we determined that diabetogenic gene(s) existed in an A/J-derived genomic interval between *D2Mit6* and *D2Mit226* (142.2 Mb interval) on chromosome 2 [4]. In this study, to narrow the genomic region containing responsible gene(s), we used newly constructed congenic strains (R1, R2, and R1A; Figure 1). The results of phenotypic analyses in these congenic strains show that at least one of responsible genes affecting obesity, hyperinsulinemia, and impaired glucose tolerance (Table 1–2, Figure 2–3) is located within a 5.8 Mb genomic interval between *D2Mit380*-*D2Mit219* (Figure 1). Because R1A mice had significantly higher body weight than SM/J mice without any difference in food intake, responsible gene(s) causes the impaired glucose tolerance without affecting the energy intake.

Within the 69.6–75.4 Mb (R1A) region on mouse chromosome 2, several QTLs for serum glucose concentration, serum insulin concentration, and glucose tolerance have been mapped by using other type 2 diabetes mouse models. In BTBR mice, *t2dm3* was mapped as a QTL controlling fasting plasma insulin and glucose concentrations [6] (Figure 1). In a subsequent study, a QTL for body mass (*Moo1*, modifier of obese 1) in BTBR mice was mapped on the interval of *t2dm3* [7]. And a more recent study using Moo1-C congenic mice suggested that gene(s) underlying the *Moo1* obesity QTL lie within the 5.7 Mb genomic interval between 71.5 Mb and 77.2 Mb [8] (Figure 1). The genomic interval of *Moo1* also overlapped with that of the R1A congenic region [7]. The *Moo1* QTL acts by increasing food intake without affecting energy expenditure, although *T2dm2sa* acts without affecting food intake (Table 2 and Figure 3). Therefore, we consider that responsible gene(s) sought in the present study may be different from the responsible gene for *Moo1*. In TSOD mice, Izumi et al. have reported that *Nidd5* for plasma insulin levels during IPGTT was mapped within a broad interval between 58.2 and 105.0 Mb [9] (Figure 1). They narrowed the locus to the 9.4 Mb interval between 57.3 and 66.7 Mb in a study using congenic mice [10], and identified the responsible gene, activin receptor-like kinase 7, located on 58.1 Mb [11]. In addition, Schmidt et al. [12] performed a meta-analysis of QTLs for diabetes-related traits and revealed that the 52–78 Mb region on mouse chromosome 2 was a consensus region with significant linkage to serum insulin concentration, glucose tolerance, body weight, and fat weight (Figure 1). These reports suggested that several diabetogenic genes exist in close proximity to our R1A region on mouse chromosome 2. We speculate that several responsible genes for *T2dm2sa* exist in genomic regions other than the R1A region, because the LOD score curve for *T2dm2sa* was broad in the QTL analysis [4]. This study shows that the R1A genomic region contains one of responsible genes for *T2dm2sa*.

To extract candidate genes, we performed an exome sequencing analysis in the R1A congenic region and identified four non-synonymous genes (*Itga6, Zak*, *Gpr155*, and *Mtx2*). *Itga6* encodes the α6-integrin subunit, which is a member of the integrin superfamily. The α6-integrin subunit is a 140-kDa protein, and heterodimerizes with a β1- or β4-subunit to function as a laminin receptor [13], [14]. The *Itga6*-knockout mice showed the absence of hemidesmosomes, severe skin blistering, and early neonatal death [15]. *Zak* encodes leucine-zipper and a sterile-alpha motif kinase, which has a serine/threonine kinase catalytic domain and activates the MAPK cascade. In the human hepatoma cell line, the overexpression of ZAK leads to the activation of the JNK/SAPK and NF-κB pathways [16]. The position of the amino acid substitution found in this study (Asn518Ser, Table 3) does not overlap with the kinase catalytic domain (17–260aa), leucine-zipper motif (280–328aa), or sterile-alpha motif domain (336–410aa). The *Gpr155* mRNA is widely expressed in adult mouse tissues and during development, but the function of the Gpr155 protein has not been elucidated [17]. The metaxin 2 protein, a mitochondrial outer membrane protein, is expressed in the mouse liver, kidney, lung, and heart. The metaxin 2 protein is located on the cytosolic face of the mitochondrial outer membrane and may play a role in protein import into mitochondria [18]. The amino acid residue (Ile) at 188 in Gpr155 is conserved among mammals as shown in Table 3. In addition, in the SNP (c.973A>G) of the *Gpr155* gene, an A/J allele (A: Ile) was also found in the C57BL/6 and LG/J strains, but SM/J allele (G: Val) was not seen in other mouse strains. These data predicted that the SM/J allele (G: Val) in the c.973A>G SNP of *Gpr155* is a relatively rare allele. Unfortunately, we could not estimate the effect of this rare allele on the Gpr155 protein function, because the Gpr155 protein function has not been elucidated. A future study is needed to investigate whether this rare variant could act as a protective allele against high-fat diet-induced obesity/diabetes. In contrast, both alleles at the other three SNPs in the *Itga6*, *Zak*, and *Mtx2* have been reported in several mouse strains and are not conserved among mammals (Table 3), which mean these SNPs are common variants and polymorphisms, rather than mutations. Knockout mouse models of four genes, which we extracted as candidate genes, do not display any overt diabetic phenotype. However, SMXA-5 and R0 original congenic mice, our animal models, develop high-fat diet-induced type 2 diabetes with the combination of diabetogenic genes. Therefore, we think that the effect of a single knockout of extracted candidate genes may be insufficient to reveal obvious phenotypes of diabetes.

Our previous data showed that neither SM/J nor A/J mice were diabetic strains, and that the diabetic phenotype in SM.A-*T2dm2sa* congenic mice was uncovered by the interaction between *T2dm2sa* and another diabetic QTL [4]. Therefore, we consider that these variants between the SM/J and A/J strains within the R1A genomic region interact with variants existing on another diabetic locus, leading to the development of diabetes. Moreover, not only rare variant SNPs (in the *Gpr155*) but also common variants (in the *Itga6*, *Zak*, and *Mtx2*) could be candidate genes for type 2 diabetes caused by the gene-gene interaction. We need to reveal whether amino-acid-altering SNPs lead to changes of these “protein functions” or not. Specifically, we have to develop functional analyses of these genes by their overexpression and/or knockdown in mammalian cells.

In conclusion, this study using R1A congenic mice demonstrated that at least one of responsible genes for high-fat diet-induced diabetes exists in the 5.8 Mb genomic interval (69.6–75.4 Mb) on chromosome 2. Moreover, within this interval, four genes with non-synonymous mutation between SM/J and A/J mice were extracted as candidate genes by exome sequencing.

## Supporting Information

Table S1 Allele variants on chromosome 2 (69.6–75.4 Mb) in A/J strain.
(PDF)

Table S2 Allele variants on chromosome 2 (69.6–75.4 Mb) in SM/J strain.
(PDF)
