# Supplementary material for: Searching for Genomic Region of High-Fat Diet-Induced Type 2 Diabetes in Mouse Chromosome 2 by Analysis of Congenic Strains
Source: PLoS One. 2014 May 1;9(5):e96271. doi: 10.1371/journal.pone.0096271 (PMC4006839; doi:10.1371/journal.pone.0096271)
Supplement: Table S1 — Allele variants on chromosome 2 (69.6–75.4 Mb) in A/J strain. (PDF) [file pone.0096271.s001.pdf]

Table S1. Allele variants on chromosome 2 (69.6-75.4 Mb) in A/J strain.

Chr	Start	Homo/Homo	SNP	Ref	Allele1	Allele2	Ref Coverage	Mut Coverage	Total Coverage	Deciebel Score	Effect	Position	Gene Symbol	strain
chr2	69540437	Homozygous	SNP	T	C	C	1	13	14	477	non_synonymous	I548V	Fastkd1	A
chr2	69540459	Homozygous	SNP	C	A	A	1	13	14	525	synonymous		Fastkd1	A
chr2	69540666	Homozygous	SNP	T	G	G	0	23	23	884	non_synonymous	Q471H	Fastkd1	A
chr2	69540751	Homozygous	SNP	T	C	C	0	28	28	1000	non_synonymous	Y443C	Fastkd1	A
chr2	69540933	Homozygous	SNP	A	G	G	0	7	7	274	splicing	IVS-27	Fastkd1	A
chr2	69545695	Homozygous	SNP	A	G	G	0	11	11	440	non_synonymous	I200T	Fastkd1	A
chr2	69550470	Homozygous	SNP	G	C	C	0	21	21	834	non_synonymous	L17V	Fastkd1	A
chr2	69574012	Homozygous	SNP	G	T	T	0	12	12	479	splicing	IVS-34	Ppig	A
chr2	69574160	Homozygous	SNP	C	T	T	1	25	26	1000	synonymous		Ppig	A
chr2	69587341	Homozygous	SNP	T	G	G	0	8	8	320	non_synonymous	N387K	Ppig	A
chr2	69619920	Homozygous	SNP	C	T	T	0	24	24	943	splicing	IVS-31	4930578N16Rik	A
chr2	69634296	Homozygous	SNP	T	C	C	0	11	11	417	3'UTR		Phospho2	A
chr2	69634901	Homozygous	SNP	A	T	T	0	10	10	400	3'UTR		Phospho2	A
chr2	69634952	Homozygous	SNP	G	T	T	0	8	8	319	3'UTR		Phospho2	A
chr2	69666882	Homozygous	SNP	G	A	A	0	11	11	440	splicing	IVS-21	Klhl23	A
chr2	69718900	Homozygous	SNP	G	A	A	0	19	19	724	synonymous		Mettl5	A
chr2	69719032	Homozygous	SNP	C	T	T	0	11	11	437	splicing	IVS-27	Mettl5	A
chr2	69719470	Homozygous	SNP	T	T	A	6	16	22	311	splicing	IVS-2	Mettl5	A
chr2	69821206	Homozygous	SNP	A	G	G	0	25	25	961	non_synonymous	N1269S	Ubr3	A
chr2	69847147	Homozygous	SNP	G	A	A	0	17	17	671	splicing	IVS-13	Ubr3	A
chr2	70082861	Homozygous	SNP	T	C	C	0	7	7	280	splicing	IVS-43	Myo3b	A
chr2	70083412	Homozygous	SNP	A	T	T	0	15	15	600	non_synonymous	T596S	Myo3b	A
chr2	70089898	Homozygous	SNP	T	C	C	0	16	16	640	synonymous		Myo3b	A
chr2	70127606	Homozygous	SNP	C	A	A	0	30	30	1000	synonymous		Myo3b	A
chr2	70188023	Homozygous	SNP	T	C	C	1	8	9	307	non_synonymous	L1227P	Myo3b	A
chr2	70365511	Homozygous	SNP	G	A	A	0	8	8	320	synonymous		4933404M02Rik	A
chr2	70805132	Homozygous	SNP	A	G	G	0	11	11	410	synonymous		Mettl8	A
chr2	70805243	Homozygous	SNP	A	G	G	0	10	10	395	splicing	IVS-30	Mettl8	A
chr2	70810363	Homozygous	SNP	T	C	C	0	10	10	400	synonymous		Mettl8	A
chr2	70810395	Homozygous	SNP	G	A	A	1	10	11	387	splicing	IVS-5	Mettl8	A
chr2	70819906	Homozygous	SNP	C	T	T	1	48	49	1000	splicing	IVS+6	Mettl8	A
chr2	70820007	Homozygous	SNP	G	A	A	1	37	38	1000	non_synonymous	R161W	Mettl8	A
chr2	70820068	Homozygous	SNP	T	C	C	0	30	30	1000	non_synonymous	T140K	Mettl8	A
chr2	70894511	Homozygous	SNP	A	G	G	0	29	29	1000	splicing	IVS-41	Dcaf17	A
chr2	70898383	Homozygous	SNP	A	C	C	0	10	10	400	splicing	IVS-27	Dcaf17	A
chr2	70916215	Homozygous	SNP	C	T	T	0	16	16	640	synonymous		Dcaf17	A
chr2	70920020	Homozygous	SNP	T	C	C	1	12	13	433	synonymous		Dcaf17	A
chr2	70920107	Homozygous	SNP	T	C	C	0	19	19	732	synonymous		Dcaf17	A
chr2	70926376	Homozygous	DEL	T	-	-	2	6	8	206	splicing	IVS-28	Dcaf17	A
chr2	70926442	Homozygous	SNP	A	G	G	0	13	13	475	synonymous		Dcaf17	A
chr2	70926547	Homozygous	SNP	G	A	A	0	6	6	240	synonymous		Dcaf17	A
chr2	70967690	Homozygous	SNP	T	A	A	0	15	15	600	splicing	IVS-20	Cybrd1	A
chr2	70967780	Homozygous	SNP	G	A	A	0	16	16	636	synonymous		Cybrd1	A
chr2	70967825	Homozygous	SNP	T	A	A	0	18	18	701	synonymous		Cybrd1	A
chr2	70976404	Homozygous	SNP	A	G	G	0	12	12	480	non_synonymous	H188R	Cybrd1	A
chr2	71087320	Homozygous	SNP	A	G	G	0	16	16	639	splicing	IVS-46	Dync1i2	A
chr2	71100873	Homozygous	SNP	C	T	T	0	9	9	332	3'UTR		Dync1i2	A
chr2	71113227	Homozygous	SNP	T	C	C	0	9	9	352	synonymous		Slc25a12	A
chr2	71113347	Homozygous	SNP	G	A	A	0	11	11	440	synonymous		Slc25a12	A
chr2	71114610	Homozygous	SNP	T	A	A	0	41	41	1000	splicing	IVS-31	Slc25a12	A
chr2	71120656	Homozygous	SNP	C	T	T	1	23	24	843	synonymous		Slc25a12	A
chr2	71120674	Homozygous	SNP	G	A	A	0	14	14	560	splicing	IVS-7	Slc25a12	A
chr2	71146232	Homozygous	SNP	A	C	C	1	9	10	355	splicing	IVS-35	Slc25a12	A
chr2	71149570	Homozygous	SNP	T	A	A	0	14	14	549	synonymous		Slc25a12	A
chr2	71153211	Homozygous	SNP	G	C	C	0	17	17	566	splicing	IVS-25	Slc25a12	A
chr2	71171784	Homozygous	SNP	C	T	T	0	11	11	414	splicing	IVS-7	Slc25a12	A
chr2	71248311	Homozygous	SNP	T	C	C	0	15	15	600	synonymous		Hat1	A
chr2	71259301	Homozygous	SNP	C	T	T	0	13	13	508	synonymous		Hat1	A
chr2	71276806	Homozygous	SNP	C	T	T	1	16	17	600	synonymous		Hat1	A
chr2	71279333	Homozygous	SNP	A	C	C	0	17	17	680	synonymous		Hat1	A
chr2	71279398	Homozygous	SNP	T	C	C	0	14	14	556	3'UTR		Hat1	A
chr2	71279409	Homozygous	SNP	A	G	G	0	12	12	480	3'UTR		Hat1	A
chr2	71349437	Homozygous	SNP	G	A	A	0	21	21	832	splicing	IVS-32	Metap1d	A
chr2	71349508	Homozygous	SNP	T	C	C	0	12	12	476	non_synonymous	W130R	Metap1d	A
chr2	71363057	Homozygous	SNP	A	G	G	0	16	16	638	3'UTR		Metap1d	A
chr2	71368996	Homozygous	SNP	C	A	A	0	39	39	1000	splicing	IVS-18	Dlx1	A
chr2	71370506	Homozygous	SNP	C	T	T	0	13	13	516	synonymous		Dlx1	A
chr2	71370518	Homozygous	SNP	T	C	C	0	16	16	615	synonymous		Dlx1	A
chr2	71370560	Homozygous	SNP	G	A	A	0	7	7	263	synonymous		Dlx1	A
chr2	71561377	Homozygous	SNP	T	C	C	1	7	8	216	non_synonymous	C33R	Gm1631	A
chr2	71561398	Homozygous	SNP	T	C	C	1	12	13	468	non_synonymous	C40R	Gm1631	A
chr2	71561509	Homozygous	SNP	A	G	G	0	6	6	240	non_synonymous	T77A	Gm1631	A
chr2	71568787	Homozygous	SNP	T	G	G	0	7	7	280	3'UTR		Gm1631	A
chr2	71568889	Homozygous	SNP	G	C	C	0	11	11	436	3'UTR		Gm1631	A
chr2	71568960	Homozygous	SNP	T	C	C	0	10	10	399	3'UTR		Gm1631	A
chr2	71654777	Homozygous	SNP	G	A	A	0	21	21	834	synonymous		Itga6	A
chr2	71663660	Homozygous	SNP	T	G	G	0	13	13	520	synonymous		Itga6	A
chr2	71663687	Homozygous	SNP	G	A	A	0	14	14	560	synonymous		Itga6	A
chr2	71664459	Homozygous	SNP	T	C	C	0	15	15	600	synonymous		Itga6	A
chr2	71664732	Homozygous	SNP	T	A	A	0	8	8	320	splicing	IVS-48	Itga6	A
chr2	71672023	Homozygous	SNP	C	T	T	0	10	10	397	splicing	IVS-43	Itga6	A
chr2	71676285	Homozygous	SNP	T	C	C	0	19	19	760	splicing	IVS-40	Itga6	A
chr2	71676341	Homozygous	SNP	C	T	T	0	18	18	716	synonymous		Itga6	A
chr2	71676365	Homozygous	SNP	G	A	A	1	29	30	1000	synonymous		Itga6	A
chr2	71684107	Homozygous	SNP	T	G	G	0	34	34	1000	synonymous		Itga6	A
chr2	71684254	Homozygous	SNP	C	G	G	0	19	19	760	non_synonymous	L975V	Itga6	A
chr2	71713554	Homozygous	SNP	C	T	T	0	12	12	470	synonymous		Pdk1	A
chr2	71713563	Homozygous	SNP	C	T	T	0	6	6	233	synonymous		Pdk1	A
chr2	71738157	Homozygous	SNP	C	T	T	0	22	22	880	synonymous		Pdk1	A
chr2	71738189	Homozygous	SNP	T	C	C	0	10	10	400	3'UTR		Pdk1	A
chr2	71869046	Homozygous	SNP	T	C	C	0	8	8	320	splicing	IVS-44	Rapgef4	A
chr2	71872125	Homozygous	SNP	T	A	A	0	10	10	391	synonymous		Rapgef4	A
chr2	71892959	Homozygous	SNP	G	A	A	0	12	12	472	5'UTR		Rapgef4	A
chr2	72046127	Homozygous	SNP	T	A	A	0	10	10	398	splicing	IVS-16	Rapgef4	A
chr2	72046129	Homozygous	SNP	T	A	A	0	11	11	437	splicing	IVS-14	Rapgef4	A
chr2	72062997	Homozygous	MNP	TG	TG	CT	9	10	19	283	splicing	IVS-21	Rapgef4	A
chr2	72064517	Homozygous	SNP	C	A	A	0	20	20	800	splicing	IVS-15	Rapgef4	A
chr2	72072265	Homozygous	SNP	A	G	G	0	26	26	1000	splicing	IVS+6	Rapgef4	A
chr2	72079531	Homozygous	SNP	G	A	A	0	11	11	439	synonymous		Rapgef4	A
chr2	74495818	Homozygous	SNP	A	C	C	1	27	28	1000	splicing	IVS+6	Evx2	A
