# Supplementary material for: Searching for Genomic Region of High-Fat Diet-Induced Type 2 Diabetes in Mouse Chromosome 2 by Analysis of Congenic Strains
Source: PLoS One. 2014 May 1;9(5):e96271. doi: 10.1371/journal.pone.0096271 (PMC4006839; doi:10.1371/journal.pone.0096271)
Supplement: Table S2 — Allele variants on chromosome 2 (69.6–75.4 Mb) in SM/J strain. (PDF) [file pone.0096271.s002.pdf]

Table S2. Allele variants on chromosome 2 (69.6-75.4 Mb) in SM/J strain.

Chr	Start	Homo/Homo	SNP	Ref	Allele1	Allele2	Ref Coverage	Mut Coverage	Total Coverage	Deciebel Score	Effect	Position	Gene Symbol	strain
chr2	69512514	Homozygous	SNP	G	T	T	0	6	6	225.98691	non_synonymous	A404S	Kbtbd10	SM
chr2	69540437	Homozygous	SNP	T	C	C	0	10	10	399.99567	non_synonymous	I548V	Fastkd1	SM
chr2	69540666	Homozygous	SNP	T	G	G	1	18	19	696.84564	non_synonymous	Q471H	Fastkd1	SM
chr2	69540751	Homozygous	SNP	T	C	C	0	24	24	959.98956	non_synonymous	Y443C	Fastkd1	SM
chr2	69540933	Homozygous	SNP	A	G	G	0	13	13	492.97952	splicing	IVS-27	Fastkd1	SM
chr2	69545695	Homozygous	SNP	A	G	G	0	14	14	559.9939	non_synonymous	I200T	Fastkd1	SM
chr2	69550470	Homozygous	SNP	G	C	C	0	12	12	479.99478	non_synonymous	L17V	Fastkd1	SM
chr2	69574012	Homozygous	SNP	G	T	T	0	6	6	239.99739	splicing	IVS-34	Ppig	SM
chr2	69619920	Homozygous	SNP	C	T	T	0	22	22	859.98566	splicing	IVS-31	4930578N16Rik	SM
chr2	69666882	Homozygous	SNP	G	A	A	0	21	21	829.98694	splicing	IVS-21	Klhl23	SM
chr2	69719470	Homozygous	SNP	T	A	A	1	10	11	308.51004	splicing	IVS-2	Mettl5	SM
chr2	69821206	Homozygous	SNP	A	A	G	5	13	18	439.48566	non_synonymous	N1269S	Ubr3	SM
chr2	69847147	Homozygous	SNP	G	A	A	1	9	10	348.27914	splicing	IVS-13	Ubr3	SM
chr2	70082861	Homozygous	SNP	T	C	C	0	8	8	319.99652	splicing	IVS-43	Myo3b	SM
chr2	70083412	Homozygous	SNP	A	T	T	0	16	16	639.99304	non_synonymous	T596S	Myo3b	SM
chr2	70188023	Homozygous	SNP	T	C	C	0	7	7	279.99695	non_synonymous	L1227P	Myo3b	SM
chr2	70347722	Homozygous	SNP	G	C	C	0	6	6	239.99739	non_synonymous	C158S	4933404M02Rik	SM
chr2	70804501	Homozygous	SNP	A	G	G	0	7	7	279.99695	splicing	IVS+4	Mettl8	SM
chr2	70805243	Homozygous	SNP	A	G	G	0	8	8	319.99652	splicing	IVS-30	Mettl8	SM
chr2	70819906	Homozygous	SNP	C	T	T	0	44	44	1000	splicing	IVS+6	Mettl8	SM
chr2	70820007	Homozygous	SNP	G	A	A	0	25	25	988.98413	non_synonymous	R161W	Mettl8	SM
chr2	70820068	Homozygous	SNP	T	C	C	0	20	20	794.99036	non_synonymous	T140K	Mettl8	SM
chr2	70894511	Homozygous	SNP	A	G	G	0	30	30	1000	splicing	IVS-41	Dcaf17	SM
chr2	70898383	Homozygous	SNP	A	C	C	0	8	8	306.98828	splicing	IVS-27	Dcaf17	SM
chr2	70967690	Homozygous	SNP	T	A	A	0	11	11	439.9952	splicing	IVS-20	Cybrd1	SM
chr2	70976404	Homozygous	SNP	A	G	G	0	10	10	388.99063	non_synonymous	H188R	Cybrd1	SM
chr2	71087320	Homozygous	SNP	A	G	G	0	14	14	539.9897	splicing	IVS-46	Dync1i2	SM
chr2	71114610	Homozygous	SNP	T	A	A	0	24	24	936.90247	splicing	IVS-31	Slc25a12	SM
chr2	71120674	Homozygous	SNP	G	A	A	0	6	6	239.99739	splicing	IVS-7	Slc25a12	SM
chr2	71146232	Homozygous	SNP	A	C	C	0	8	8	317.99628	splicing	IVS-35	Slc25a12	SM
chr2	71153211	Homozygous	SNP	G	C	C	0	15	15	525.9321	splicing	IVS-25	Slc25a12	SM
chr2	71171784	Homozygous	SNP	C	T	T	1	16	17	645.8613	splicing	IVS-7	Slc25a12	SM
chr2	71349437	Homozygous	SNP	G	A	A	0	22	22	862.9855	splicing	IVS-32	Metap1d	SM
chr2	71349508	Homozygous	SNP	T	C	C	0	20	20	789.9874	non_synonymous	W130R	Metap1d	SM
chr2	71368996	Homozygous	SNP	C	A	A	1	31	32	1000	splicing	IVS-18	Dlx1	SM
chr2	72236298	Homozygous	SNP	C	A	A	1	7	8	257.01877	splicing	IVS-49	B230120H23Rik	SM
chr2	72250081	Homozygous	SNP	T	C	C	4	28	32	964.99054	splicing	IVS-47	B230120H23Rik	SM
chr2	72273267	Homozygous	SNP	A	G	G	1	17	18	634.9924	splicing	IVS-21	B230120H23Rik	SM
chr2	72276260	Homozygous	SNP	A	G	G	1	23	24	871.98816	non_synonymous	N518S	B230120H23Rik	SM
chr2	72321393	Homozygous	SNP	T	C	C	1	12	13	463.0823	splicing	IVS-19	Cdca7	SM
chr2	73211964	Homozygous	SNP	T	C	C	0	7	7	279.99695	non_synonymous	I188V	Gpr155	SM
chr2	73282659	Homozygous	SNP	C	G	G	0	23	23	904.9767	splicing	IVS-28	Wipf1	SM
chr2	73452868	Homozygous	SNP	T	G	G	0	12	12	463.9779	splicing	IVS-44	Chn1	SM
chr2	73456141	Homozygous	SNP	T	C	C	1	15	16	559.9939	splicing	IVS-26	Chn1	SM
chr2	73462288	Homozygous	SNP	C	A	A	0	9	9	345.99152	splicing	IVS-39	Chn1	SM
chr2	74495818	Homozygous	SNP	A	C	C	0	14	14	559.9939	splicing	IVS+6	Evx2	SM
chr2	74714508	Homozygous	SNP	T	C	C	0	10	10	399.99567	non_synonymous	W257R	Mtx2	SM
